# Audiovisual Temporal Processing in Postlingually Deafened Adults with Cochlear Implants

**DOI:** 10.1038/s41598-018-29598-x

**Published:** 2018-07-27

**Authors:** Iliza M. Butera, Ryan A. Stevenson, Brannon D. Mangus, Tiffany G. Woynaroski, René H. Gifford, Mark T. Wallace

**Affiliations:** 1 Vanderbilt Brain Institute, Vanderbilt University, Nashville, TN USA; 2 Department of Psychology, University of Western Ontario, London, ON Canada; 3 Brain and Mind Institute, University of Western Ontario, London, ON Canada; 4 Murfreesboro Medical Clinic and Surgicenter, Murfreesboro, TN USA; 5 Department of Hearing and Speech Sciences, Vanderbilt University, Nashville, TN USA; 6 Vanderbilt Kennedy Center, Vanderbilt University Medical Center, Nashville, TN USA

## Abstract

For many cochlear implant (CI) users, visual cues are vitally important for interpreting the impoverished auditory speech information that an implant conveys. Although the temporal relationship between auditory and visual stimuli is crucial for how this information is integrated, audiovisual temporal processing in CI users is poorly understood. In this study, we tested unisensory (auditory alone, visual alone) and multisensory (audiovisual) temporal processing in postlingually deafened CI users (n = 48) and normal-hearing controls (n = 54) using simultaneity judgment (SJ) and temporal order judgment (TOJ) tasks. We varied the timing onsets between the auditory and visual components of either a syllable/viseme or a simple flash/beep pairing, and participants indicated either which stimulus appeared first (TOJ) or if the pair occurred simultaneously (SJ). Results indicate that temporal binding windows—the interval within which stimuli are likely to be perceptually ‘bound’—are not significantly different between groups for either speech or non-speech stimuli. However, the point of subjective simultaneity for speech was less visually leading in CI users, who interestingly, also had improved visual-only TOJ thresholds. Further signal detection analysis suggests that this SJ shift may be due to greater visual bias within the CI group, perhaps reflecting heightened attentional allocation to visual cues.

## Introduction

Cochlear implantation is an effective surgical intervention for individuals with severe-to-profound sensorineural hearing loss to either regain auditory speech perception or, for the congenitally deaf, to establish it for the first time. This highly successful neuroprosthetic device parcels acoustic signals into frequency bins that correspond to tonotopic stimulation of intracochlear electrodes. Despite considerable technological and surgical advancements, spread of electrical excitation in the cochlea remains a significant barrier for cochlear implant (CI) users to achieve high-fidelity spectral encoding. As a result, the degraded auditory signal that an implant provides can be quite ambiguous to some CI users^1–3^.

Fortunately, speech is typically an audiovisual (AV) experience wherein coincident orofacial articulations can considerably boost perceptual accuracy over that observed with auditory-alone stimulation^4^. Indeed, a great deal of modeling work suggests that ambiguous information stemming from unreliable sensory estimates is optimally integrated in the brain by weighting the relative reliability of the different sources of sensory evidence^5–7^. This process results in a more robust multisensory percept with specific advantages including increased stimulus saliency^8^, decreased detection thresholds^9,10^, reduced reaction times^11^, and enhanced efficiency in neural processing^12^.

Many of these multisensory-mediated benefits have been seen in children who have received early cochlear implantation (i.e., before age 4). These include: faster reaction times^11^, greater multisensory gain^13,14^, and higher speech recognition at multiple levels of phonetic processing^15^. Furthermore, it has been suggested that many CI users may achieve audiovisual speech recognition abilities that are comparable to normal-hearing individuals after matching unisensory performance (e.g., through masking or generating CI simulations of speech for typical listeners)^16^. In support of this claim, several studies in both CI users and other hearing-impaired populations indicate proficient multisensory integration as measured via AV speech recognition tests of consonants^17^, phonemes^18^, words^16,19,20^, and sentences^14,21^, as well as in work that employs computational models^22,23^.

The perceived timing of auditory and visual information is a core determinant in the efficacy by which cues are integrated and perceptually ‘bound’ (see below)^5,24,25^. Although the aforementioned studies illustrate that AV integration can be quite good in CI users, temporal processing is a critical factor in this process and, somewhat surprisingly, very little is known about AV temporal processing in the CI population. Thus, we questioned whether the auditory information conveyed by a cochlear implant may alter the perceptual weighting of visual and auditory cues in such a way that generalizes to differences in AV temporal assessments compared to normal-hearing individuals.

In typical development, it is well established that AV stimuli are more likely to be perceptually bound when the individual component stimuli are in temporal (as well as spatial) proximity^24^. However, AV stimuli need not be precisely synchronous for this binding to occur, but rather appear to be integrated over a range of temporal offsets spanning several hundred milliseconds, a construct known as the temporal binding window (TBW)^26^. Only one published study has investigated the temporal binding of AV stimuli in CI users, and the authors indicated no difference in TBWs between age-matched, normal-hearing (NH) individuals and CI users during the presentation of monosyllabic words^27^. Other work has reported similar findings for the moderately hearing-impaired while judging whether AV sentences were either synchronous or asychronous^28^. In contrast, few studies have investigated AV temporal function across a broader range of stimulus types ranging from the simplistic (i.e., flashes and beeps) to the more speech-related^11,29,30^. Furthermore, given the use of word and sentence stimuli in prior work, we sought to examine here whether differences in AV temporal performance would be evident in CI users while making less complex, sublexical temporal judgments.

The present study investigates multisensory (i.e., combined audiovisual) temporal processing in CI users and a group of NH controls using speech syllables and simple “flashbeep” stimuli. Because early access to sound is an important factor for the development of multisensory integration, we recruited postlingually deafened adult CI users to test whether auditory, visual, and audiovisual temporal functions are altered in those who experience typical auditory development in early life (in contrast to prelingual deafness). In normal-hearing individuals, the TBW has been shown to both narrow as well as become more asymmetrical during development^31,32^. Thus, our primary hypothesis was that CI users, given their altered AV experience, would exhibit broader temporal binding windows centered closer to objective simultaneity (i.e., 0 ms) than controls. Practically, this would mean CI users are less able to accurately identify AV asynchronies. We expected this result to be specific for speech stimuli and not for simple flashbeep stimuli on account of the greater ecological validity of speech signals. We drew this prediction in part from prior work investigating the maturation of temporal binding windows in normal development^32^, and reasoned that reduced auditory experience during deafness might result in less mature (i.e., broader and more symmetric) temporal binding windows to be evident well into adulthood for CI users.

**Figure 1:**
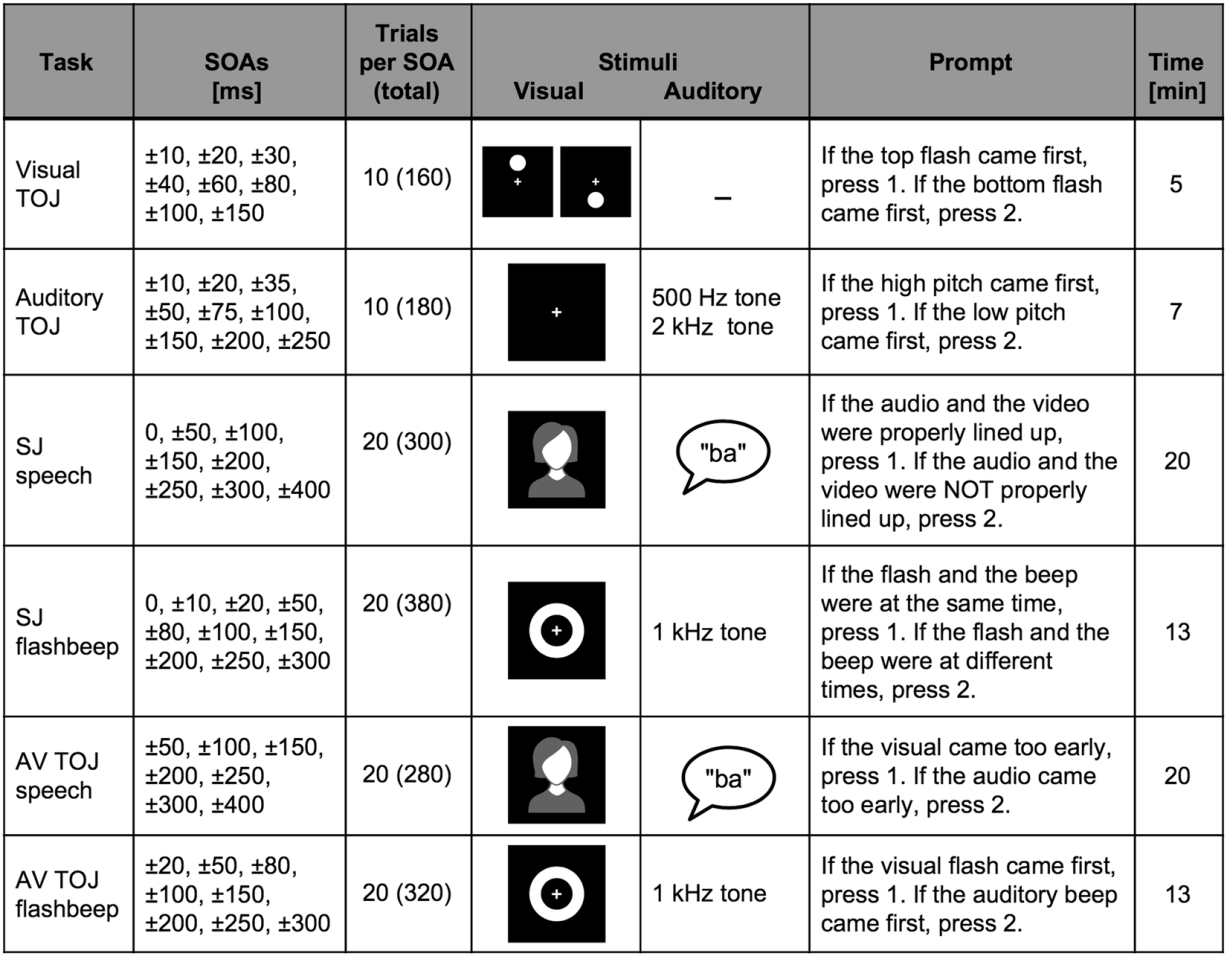
Psychophysical tasks included unisensory and multisensory conditions for the temporal order judgment (TOJ) and simultaneity judgment (SJ) of either circles and tones or speech. SOA = stimulus onset asynchrony.

**Figure 2:**
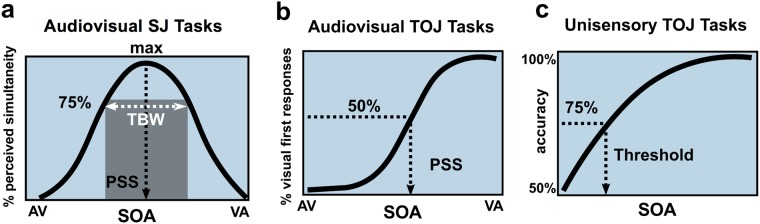
Summary metrics by task. For SJ tasks (**a**), the percent of perceived simultaneity is plotted per SOA from auditory-leading to visually-leading offsets in order to derive TBW at 75% and the PSS from the peak of the function. Two opposing logit functions are used for TBW curve fits so that symmetry is not assumed, while Gaussian functions are used for PSS derivation. For AV TOJ tasks (**b**), the percent of visual-first responses is plotted, and PSS at 50% is calculated from the resulting sigmoid functions. For unisensory TOJ tasks, percent accuracy is plotted in order to collapse across positive and negative SOAs that are arbitrarily defined as the top circle/high pitch occurring first (+SOA) or the bottom circle/low pitch occurring first (−SOA). Threshold is derived from a logit function at 75% accuracy. SJ = simultaneity judgement; TBW = temporal binding window; SOA = stimulus onset asynchrony; PSS = point of subjective simultaneity; TOJ = temporal order judgment; AV = audiovisual.

We tested our hypothesis using two distinct tasks: simultaneity judgment (SJ) and temporal order judgment (TOJ)—the latter of which we also used to quantify unisensory temporal thresholds (Fig. 1). During SJ tasks, which are commonly used to measure TBWs, individuals are presented with auditory and visual stimuli that vary in relative synchrony and asked to report whether they perceived the two stimuli to have occurred at the “same time” or at “different times.” SJ tasks were administered using both a simple (i.e., “flashbeep”) stimulus and a more complex speech syllable stimulus presented at 12–19 different stimulus onset asynchronies (SOAs) (see Fig. 1). In a common measure closely tied to TBW, we also quantified the point of subjective simultaneity (PSS)—the SOA at which maximal reports of perceived synchrony occurred (Fig. 2a). In adults, PSS is typically visual leading (by convention represented as a positive SOA), which likely reflects an adaptation to the relative physical transmission speeds of light versus sound^24^.

Additionally, an AV TOJ task utilized the same stimuli as the SJ task but with instructions to report the stimulus order instead of the apparent synchrony. Finally, unisensory temporal processing was also assessed with auditory TOJ (aTOJ) and visual TOJ (vTOJ) tasks wherein two brief unisensory stimuli (e.g., two circles or two tones) are presented in rapid succession at varying SOAs, and individuals report which stimulus occurred first. In prior studies from our group, this testing battery has been used to evaluate temporal thresholds of unisensory and multisensory processing of simple stimuli and speech syllables in typical populations across the lifespan^31–33^ and in individuals with neurodevelopmental disorders^34–36^. It is employed here using similar methods to evaluate adult postlingually deafened CI users in comparison to NH controls.

## Results

Using nonparametric (i.e., bootstrapped) univariate regressions, we performed planned analyses of between-group differences across eight summary metrics (Fig. 2) derived from six psychophysics tasks. Group differences in age and nonverbal IQ prompted us to explore these indices as covariates (see Methods for participant characteristics); these factors were retained in all models wherein they accounted for significant variance. To further explore significant findings, post hoc follow-up tests evaluated between-group differences at all individual SOAs using multivariate analysis of variance (MANOVA) or a multivariate analysis of covariance (MANCOVA), where appropriate.

### Overview of findings for summary metrics

A statistically significant difference in performance was found between groups for SJ speech PSS, with mean values of 15.5 ms in the CI group and 54.7 ms in the NH controls (p = 0.004, Table 1). Additionally, a significant between-group difference was observed in vTOJ thresholds when controlling for age and nonverbal IQ (which were significant predictors in the vTOJ regression model). Interestingly, CI users had improved thresholds relative to NH controls (corrected means = 38.1 ms and 49.5 ms, respectively; p = 0.004).

**Table 1:** Between-subjects univariate regressions including covariates where indicated by an asterisk. Significance was set at α = 0.05, and bolded values met this threshold.

Psychophysical tasks	F statistic	Significance	Effect size (partial η^2^)
SJ flashbeep PSS	F_(1,91)_ = 1.9	p = 0.17	0.021
SJ flashbeep TBW	F_(1,85)_ = 0.47	p = 0.49	0.006
SJ speech PSS	F_(1,97)_ = 8.8	**p = 0.004**	0.084
SJ speech TBW	F_(1,88)_ = 0.96	p = 0.33	0.011
Visual TOJ threshold*	F_(1,82)_ = 8.6	**p = 0.004**	0.095
Auditory TOJ threshold	F_(1,77)_ = 1.2	p = 0.28	0.015
avTOJ flashbeep PSS	F_(1,74)_ = 0.33	p = 0.57	0.004
avTOJ speech PSS	F_(1,57)_ = 3.7	p = 0.06	0.060

TOJ = temporal order judgment; SJ = simultaneity judgment, TBW = temporal binding window; PSS = point of subjective simultaneity.

Thus, the CI group has: 1) PSS for speech that is shifted *away* from visually-leading SOAs (i.e., less positive) and 2) improved visual temporal thresholds. Between-group differences were not observed for any other measures; however, PSS for the audiovisual speech TOJ task was noteworthy in its marginally significant group differences (p = 0.06, Table 1) between CI users and controls (−78.2 ms and −7.83 ms, respective means), which is consistent with the PSS shift observed in response to the SJ speech task.

### Perceived synchrony of speech stimuli is less visually leading in CI users

**Figure 3:**
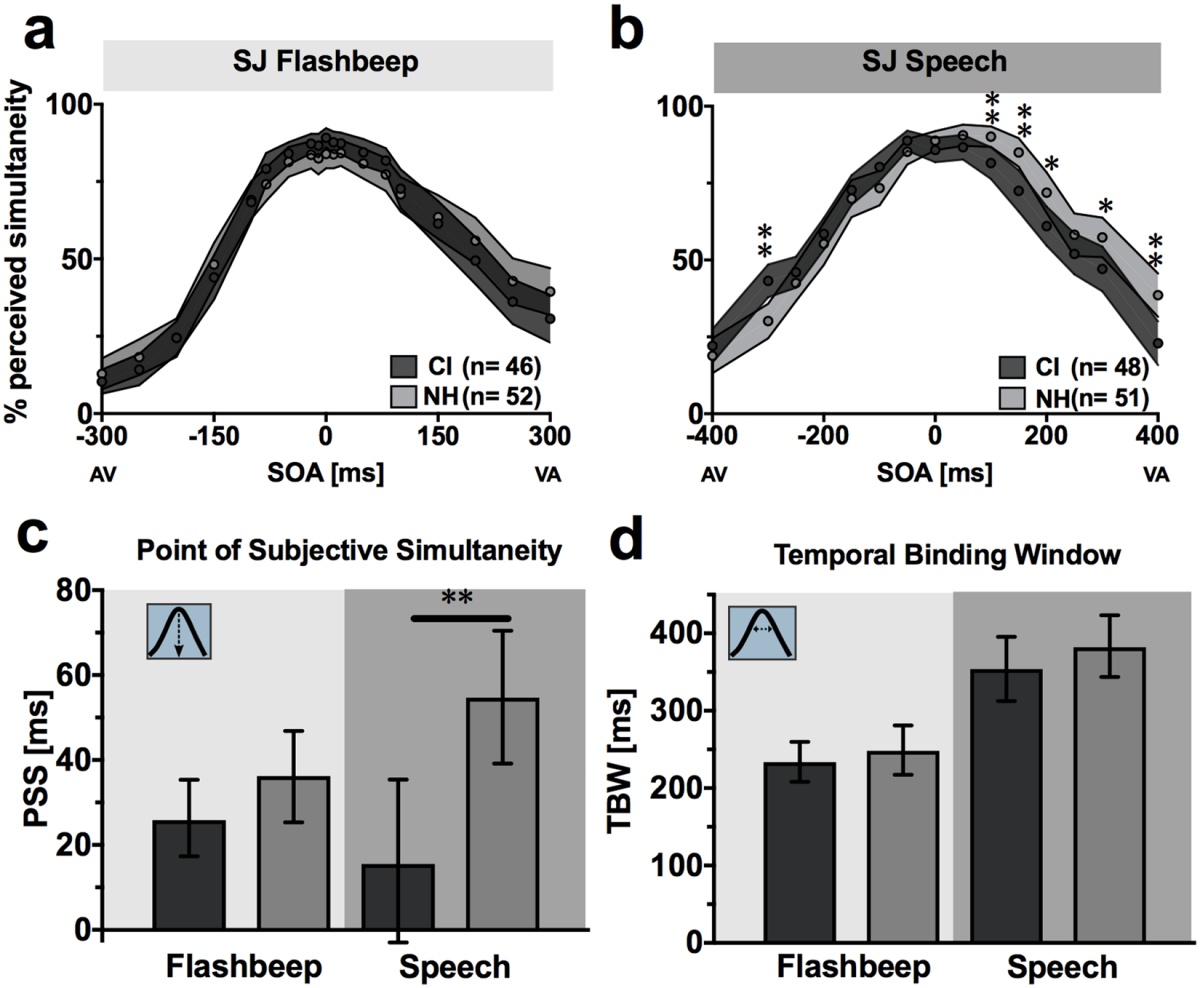
Simultaneity judgment. Mean reports of simultaneity at each SOA are plotted for flashbeep (**a**) and speech tasks (**b**). CI users are shown in dark gray and NH in light gray. Circles indicate means and shaded areas correspond to 95% confidence interval of the mean. Mean PSS (**c**) and TBW (**d**) calculations are shown with error bars also indicating 95% confidence intervals. CI = cochlear implant; NH = normal hearing; SOA = stimulus onset asynchrony; TBW = temporal binding window; PSS = point of subjective simultaneity; *p < 0.05, **p < 0.01.

Audiovisual temporal function was examined using the SJ task wherein we varied the timing onset between the auditory and visual components of either a syllable/viseme or a less complex circle/beep pairing and had participants indicate if the pair appeared synchronous. As illustrated in Fig. 3a,b, performance is plotted as mean reports of synchrony for each SOA. Not surprisingly, for the flashbeep task (Fig. 3a), confidence intervals are highly overlapping between CI users and NH controls. This plot further supports the aforementioned lack of group difference in AV temporal acuity for low-level stimuli (Table 1), which requires no further follow-up testing. In contrast, a post hoc MANOVA for the speech task (dependent variables: 15 SOAs) indicated a statistically significant difference between groups (F_(15,83)_ = 3.05, p = 0.001; Wilk’s Λ = 0.645, η_p_^2^ = 0.355). Follow-up univariate tests indicated that the CI group differed from the NH controls at six (out of 15) SOAs. These include the following negative (i.e., auditory-leading) asynchronies and positive (i.e., visually-leading) asynchronies: −300 ms (F_(1,97)_ = 11.3, p = 0.001, η_p_^2^ = 0.11), 100 ms (F_(1,97)_ = 8.009, p = 0.006, η_p_^2^ = 0.076), 150 ms (F_(1,97)_ = 9.66, p = 0.002, η_p_^2^ = 0.091), 200 ms (F_(1,97)_ = 5.62, p = 0.02, η_p_^2^ = 0.055), 300 ms (F_(1,97)_ = 4.49, p = 0.037, η_p_^2^ = 0.044), and 400 ms (F_(1,97)_ = 9.84, p = 0.002, η_p_^2^ = 0.092). These differences appear to be a result of group-level differences in PSS, which was derived from individual curve fits then averaged across groups (Fig. 2a). Thus, the overall shift to the left (toward 0 ms) in CI users relative to NH subjects is evident both in the individual PSS calculations (Fig. 3c) and the averaged responses at each SOA (Fig. 3b).

Contrary to our hypothesis, there were no significant differences in TBW for either the flashbeep or speech stimuli (Table 1; Fig. 3d). Collectively, these results indicate a shift in AV temporal performance in CI users that is specific for speech stimuli, and that manifests not as a change in overall temporal acuity (i.e., a TBW shift), but rather as a shift in the peak of this Gaussian function toward objective synchrony (i.e., a PSS shift toward an SOA of 0 ms).

### TOJ of multisensory speech further supports a shift in PSS between groups

**Figure 4:**
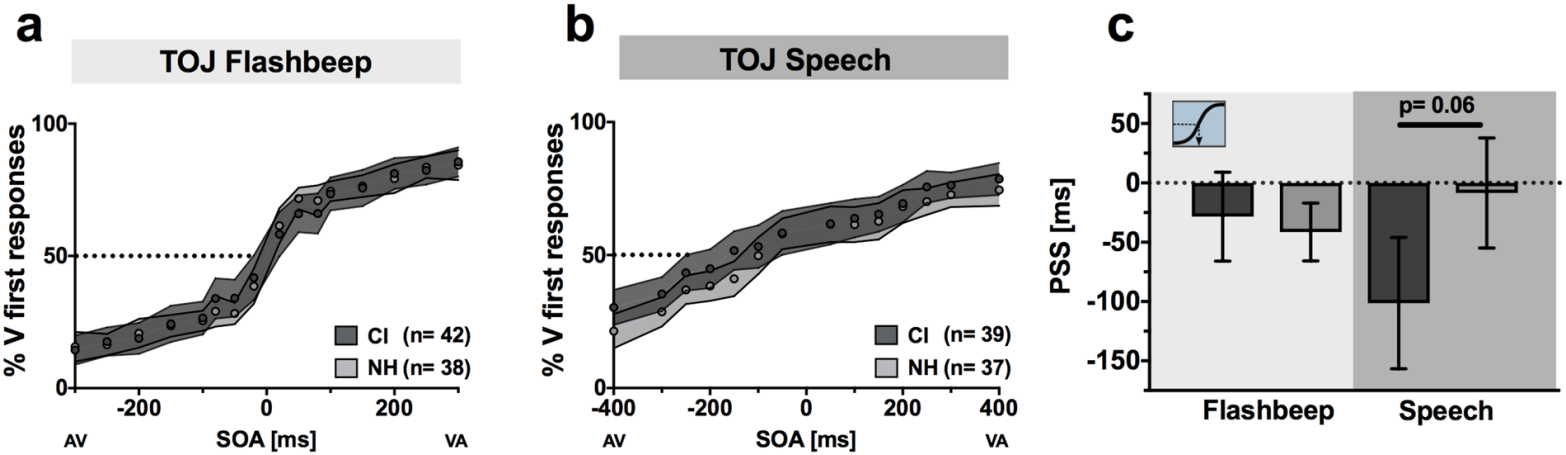
Audiovisual TOJ tasks. Mean percent of “visual-first” responses per SOA are plotted for audiovisual flashbeep (**a**) and speech (**b**) TOJ tasks. CI users are shown in dark gray and NH in light gray. Circles indicate means, and shaded areas correspond to 95% confidence intervals of the mean. PSS (point of subjective simultaneity; (**c**) for both tasks were derived for each subject and averaged across groups by the intersection of the psychometric function with 50%. Error bars indicate 95% confidence intervals. CI = cochlear implant; NH = normal hearing; SOA = stimulus onset asynchrony.

In order to further explore the shift in PSS for CI users, we tested AV temporal function using AV TOJ tasks. With the same stimuli from the SJ tasks, individuals were instructed to indicate whether the visual or auditory component occurred first. The proportion of “visual first” responses is plotted for each SOA in the flashbeep (Fig. 4a) and speech task (Fig. 4b). The PSS for TOJ is calculated as the SOA at which the two alternatives (auditory first or visual first) are equally likely. It should be noted that maximum perception of synchrony (SJ PSS) is similar but not equivalent to the maximum uncertainty of the presentation order (TOJ PSS)^37–39^, because it is possible to perceive two objects as asynchronous but to still be unsure of which occurred first. Accordingly, the PSS values for the TOJ tasks (Fig. 4c) differ in magnitude compared to the SJ tasks (Fig. 3c), yet appear to support the result that CI users differ for PSS only with the speech tasks. Although temporally shifted judgments of speech TOJ were not statistically significant (Table 1), the overall pattern is consistent with the SJ task and more importantly, also provide an opportunity to further investigate underlying decisional biases in AV temporal judgments using signal detection theory (SDT) derived analyses.

### Signal detection analysis of the audiovisual speech TOJ task reveals a visual response bias in CI users

After pooling responses from all subjects in each group for all four TOJ tasks, we applied signal detection methods to calculate measures of sensitivity (d′) and response bias (c) (see Methods). For these measures, larger d′ values correspond to higher sensitivity or discriminability, and bias values of zero represent an “unbiased” response (i.e., one in which the criterion is unchanged). For the AV TOJ tasks, we calculated the probability of correct “visual first” responses to positive SOAs (i.e., VA trials) and incorrect “visual first” response to negative SOAs (i.e., AV trials). Non-overlapping confidence intervals are considered significant group differences at the corresponding SOAs for both sensitivity and bias.

**Figure 5:**
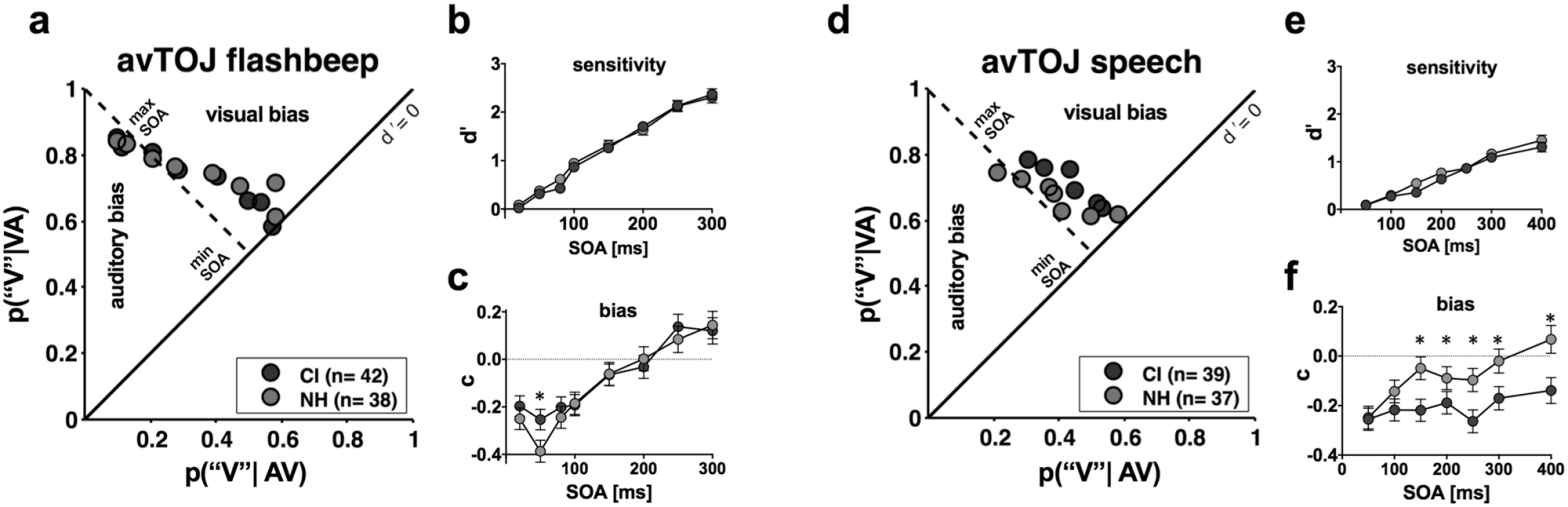
Audiovisual TOJ tasks. The probability of hits versus probability of false alarms are plotted for the audiovisual TOJ flashbeep (**a**) and audiovisual TOJ speech tasks (**d**). From these ROC plots, sensitivity (d′) and response bias (c) were calculated for each SOA and plotted for the flashbeep (**b**,**c**) and speech stimuli (**e**,**f**), respectively. Error bars indicate 95% confidence intervals of the mean. * = non-overlapping confidence intervals.

For the AV TOJ flashbeep task (Fig. 5a), the ROC plot reveals that there are nearly equivalent shifts in sensitivity between groups (Fig. 5b). Thus, there are no differences between groups for d′, although the lower overall sensitivity for speech TOJ (Fig. 5e) compared with flashbeep TOJ (Fig. 5b) illustrates the comparatively greater difficulty of the speech task (i.e., maximum d′ of 1.5 v. 2.4). Furthermore, bias appears comparable between the two groups, except for one significant difference at the 50 ms SOA (Fig. 5c). In contrast, for the AV TOJ speech task, there are apparent differences in the ROC plot (Fig. 5d) such that CI users have more ‘visual first responses.’ Again, there are no differences in sensitivity between groups (Fig. 5e); however, response bias differs significantly between groups for all but the two shortest SOAs (50 and 100 ms). This finding reflects a visual bias for CI users toward a greater likelihood of selecting the ‘visual first’ response (Fig. 5f). In summary, data from these AV TOJ tasks reveal strikingly similar performance between groups for the flashbeep task, similar sensitivity for the speech task, and substantially different response biases for the speech task.

### Visual TOJ thresholds are improved in CI users, but auditory thresholds do not significantly differ from controls

**Figure 6:**
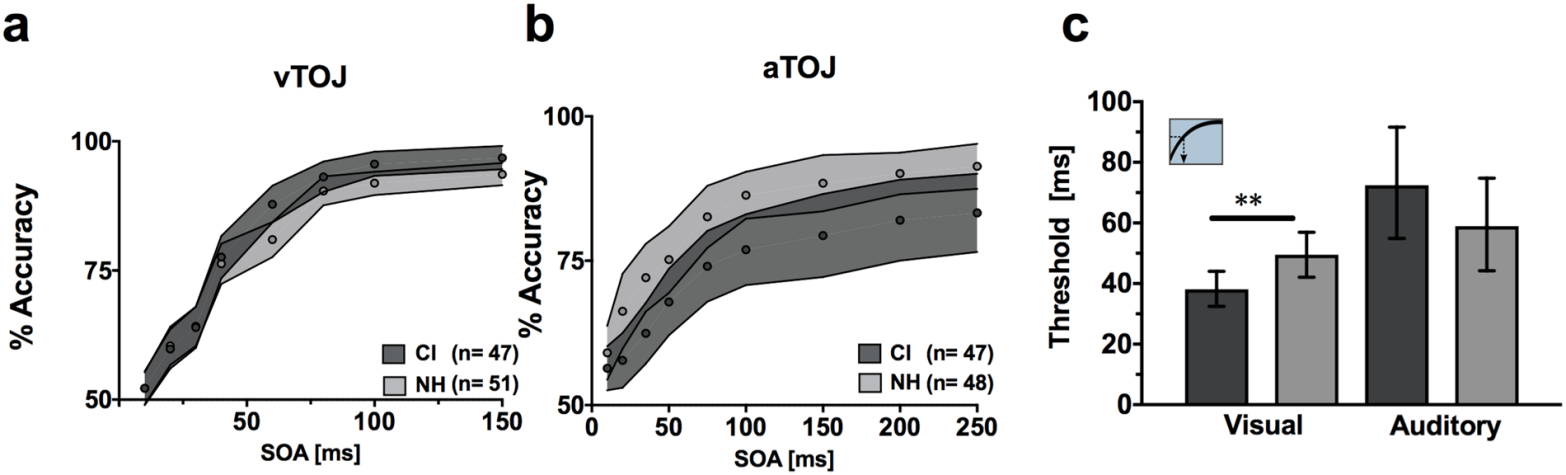
Unisensory TOJ tasks. Mean accuracies per SOA are plotted for visual (**a**) and auditory (**b**) TOJ tasks. CI users are shown in dark gray and NH in light gray. Circles indicate means and shaded areas correspond to 95% confidence intervals. Threshold calculations (**c**) at 75% accuracy are shown for both tasks with error bars also indicating 95% confidence intervals. CI = cochlear implant; NH = normal hearing; SOA = stimulus onset asynchrony; aTOJ = auditory temporal order judgment; vTOJ = visual temporal order judgment; **p < 0.01.

To illustrate temporal unisensory performance across groups, we plotted performance at each tested SOA for the CI users and NH controls. Group averages for visual TOJ (a) and auditory TOJ (b) are plotted in Fig. 6. As noted previously (Table 1), thresholds measured at 75% accuracy were significantly different between groups for the visual TOJ task but not the auditory TOJ task (Fig. 6c). A post hoc MANCOVA for the vTOJ task (dependent variables: 8 SOAs, covariate: age) did not indicate any further statistically significant group differences (F_(8,88)_ = 1.74, p = 0.1; Wilk’s Λ = 0.863, η_p_^2^ = 0.137). Therefore, significant group differences in vTOJ are limited to threshold measures (Fig. 6c) and not any broader differences across individual SOAs (Fig. 6a). Because a difference in threshold could result from either differences in low level sensory processing (i.e., sensitivity or discriminability changes) or higher level decisional factors (i.e., bias or criterion changes), we further investigated these results using SDT.

### Signal detection analysis reveals differences in aTOJ sensitivity and vTOJ response bias

**Figure 7:**
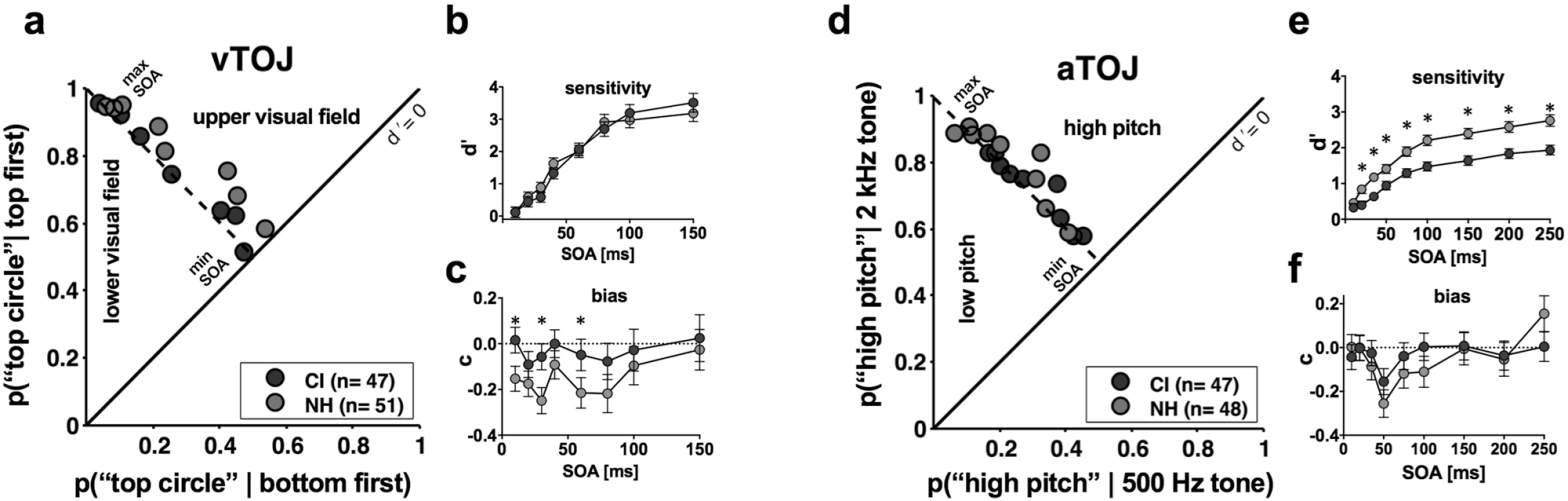
Auditory and Visual TOJ tasks. The probability of hits versus probability of false alarms are plotted for the vTOJ (**a**) and aTOJ tasks (**d**). From these ROC plots, sensitivity (d′) and response bias (c) were calculated for each SOA and plotted for vTOJ (**b,c**) and aTOJ (**e,f**), respectively. Error bars indicate 95% confidence intervals of the mean. * = non-overlapping confidence intervals.

The probability of correct responses to negative SOAs is plotted on the y axis versus incorrect responses to positive SOAs on the x axis in ROC space for both the visual (Fig. 7a) and auditory TOJ tasks (Fig. 7d). For the visual task, negative SOAs were arbitrarily defined as trials where the top circle appeared first, and for the auditory task, when the high pitch was presented first. For the vTOJ task we see equivalent sensitivity (Fig. 7b) yet a shift in response bias such that CI users are more like unbiased observers (dashed line) compared to NH controls. Curiously, this difference in the NH group manifests as a bias in reporting the stimulus in the upper visual field appearing first (Fig. 7c). For the aTOJ task (Fig. 7d), sensitivity is lower in the CI group at all but the most difficult SOA (Fig. 7e). In contrast, response bias is unaffected in the aTOJ task (Fig. 7f). Together, these findings suggest that: 1) CI users employ distinct response strategies in the vTOJ task that minimize response biases, and 2) CI users have decreased sensitivity for the aTOJ task.

## Discussion

**Figure 8:**
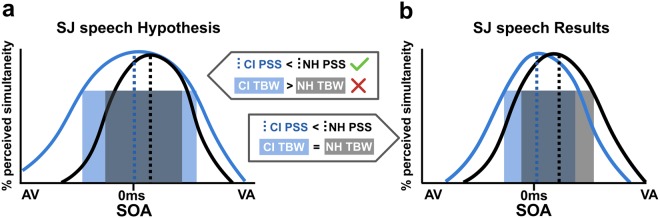
Summary of main hypothesis and results. Although the direction of our hypothesized speech PSS shift (**a**) was supported by our results (**b**), the speech TBW for CI users was non-significantly different from controls and not extended as we had anticipated.

A key finding in this study is a shift in the point of subjective simultaneity (PSS) for making temporal judgments regarding audiovisual speech in postlingually deafened adults with CIs compared to NH controls. Specifically, the NH control group required visual speech cues to precede auditory speech cues by 39 ms more than CI users at the point of maximum perceived simultaneity in the SJ task (Fig. 3c). This finding is consistent with our expectation of a PSS more centered near objective synchrony; however, we expected this shift to be accompanied by a broadening of the overall width of the temporal binding window for speech stimuli (Fig. 8a), a result that was not supported by the data. Rather, CI users had lower reports of simultaneity at auditory-leading speech SOAs (Fig. 3b), resulting in comparable TBWs for both groups (Fig. 8b). Thus, compared to NH controls, CI users have greater accuracy identifying temporal asynchronies with visual-leading speech, yet lower accuracy with auditory-leading speech.

We first questioned how any artificial auditory latency introduced by CI processors may have influenced our group-level differences in speech PSS. Although exact processing times vary by the manufacturer, sound processor, and programmed pulse rate, the magnitude is generally on the order of 10 ms. Because this processing is bypassing acoustic conduction in the middle and inner ear, actual first spike latencies in the VIII cranial nerve in CI users compared to NH controls would actually differ by less than 10 ms. As a result, SOAs in our study may have an artificial auditory delay of at most 10 ms in the CI group. If that is the case, then SOAs for CI users are actually *more* visually-leading, which is rightward and in the opposite direction of our findings of *less* visually-leading subjective synchrony in CI users (i.e., leftward shifts closer to objective synchrony at 0 ms). Therefore, if greater auditory latency was introduced by CI processors, then the group-level differences in SJ speech PSS are even more robust.

Interestingly, from the only other published test of simultaneity judgments in CI users, Hay-McCutcheon *et al*. report a similar, albeit statistically non-significant trend of CI users in a similar age group having a smaller PSS (i.e., CI = 58 ms; NH = 72 ms). It is possible that their small sample (n = 12) contributed to a statistically underpowered comparison in the self-described preliminary analysis^27^. If so, the same leftward shift may exist for both syllables (shown here) as well as full words. To our knowledge, the significant speech PSS shift reported here is a novel finding and one that warrants further investigation to more conclusively establish whether broader generalization exists with other speech cues (e.g., full words and sentences).

The observed shift in PSS in the CI users could be a result of changes in so-called “bottom up” (i.e., stimulus-dependent) factors or due to changes in more “top-down” (i.e., decisional) factors such as response bias. To delve further into these differences, we utilized a different temporal paradigm – the temporal order judgment task – that allows for a dissection of these factors using principles derived from signal detection theory (SDT) and, specifically, the calculation of measures of sensitivity and response bias. Although not strictly adhering to SDT, these analyses strongly suggest no differences between groups in AV temporal sensitivity (for either flashbeep or speech stimuli), but a significant difference in response bias for the speech stimuli, manifesting as a strong visual bias for the CI users (Fig. 5f).

We then considered whether, in the absence of any prompting from the task instructions, CI users had preferentially directed their attention toward the visual speech component throughout the SJ task. The effects of such attentional cueing have been measured in a number of studies investigating attention-dependent sensory acceleration, also known as “prior entry”^40–42^. For example, endogenous cueing that overtly directs a viewer’s attention toward the visual component in an SJ task causes the PSS to shift leftward by 14 ms when stimuli are short noise bursts and illuminated LEDs^40^. Given the higher complexity of speech syllables as well as the temporal ambiguity introduced from articulation onset to voicing onset, we consider a 39 ms SJ PSS shift here as a reasonable magnitude to fall within this explanation. Interestingly, physically manipulating a visual stimulus to be more salient than an auditory cue (i.e., exogenous cueing) also shifts PSS in a similar manner^43^. Broadly speaking, highly salient, attention-grabbing stimuli of various types (i.e., either crossmodal or intramodal) are well-known to be perceived as occurring prior to less salient ones^40,44,45^. Thus, in the absence of any overt attentional cueing in the instructions or by the researchers, CI users’ SJ speech curves appear biased toward vision (fewer reports of synchrony at +SOAs) at the expense of auditory-leading trials (more reports of synchrony at − SOAs). Therefore, an attend-vision response strategy in the CI group may explain an overall leftward shift in PSS as seen in the speech SJ task without a constriction of the TBW (Fig. 8b).

This finding is novel in that it suggests that CI users are employing greater visual weighting in temporal judgments of speech (when compared with NH individuals). Thus, for CI users, low reliability of an auditory sensory estimate likely results in placing lower weight on the auditory information in the process of AV cue combination^6^. It seems plausible that daily, focused lip reading while listening with a CI causes higher perceptual weighting of the visual speech signal. Interestingly, a recent study in pediatric CI users reported lower auditory dominance for temporal judgments, and this lessened auditory weighting had a negative impact on language skills in a prelingually deafened cohort^30^.

Unlike the paucity of data surrounding AV temporal processing in CI users, perception of AV syllables has been extensively studied via a common proxy for multisensory integration called the McGurk effect^29,46^. In this crossmodal illusion, conflicting AV syllables can elicit a novel percept. For example, a sound file of the syllable “ba” dubbed onto the visual articulation of “ga” often elicits the perception of a third syllable such as “da” or “tha” for the viewer. Presentation of these incongruent stimulus pairings creates a perceptual discrepancy that drives individuals to report either the fused multisensory percept or the token for the sense providing the best sensory estimate (i.e., visual or auditory capture). Highly consistent results across several studies of the McGurk illusion indicate a visual bias in CI users that is rarely seen in NH individuals^18,47–50^. Our results here using the same syllables from the McGurk illusion (“ba” and “ga”) suggests that temporal judgements of these AV cues are also visually-biased^51^.

Turning to the unisensory TOJ tasks, we also found differences in vTOJ thresholds (Fig. 6c) and response bias between groups (Fig. 7c). A possible explanation for these differences is that the task required subjects to fixate on a cross in the center of the screen and distribute their spatial visual attention to monitor two locations in the upper and lower visual fields. It is possible that the ability of CI users to perform more like unbiased observers reflects enhanced attentional allocation to the relevant parafoveal visual locations^52^. NH controls were seemingly less able to do this, and instead focused more on the upper visual field. In the NH group, this response bias may have resulted for several plausible reasons. One possibility is that visual apparent motion may have been experienced as a result of rapidly flashing the circles in quick succession. In the absence of well-distributed visual attention, NH controls may simply have responded according to known anisotropies in visual apparent motion detection to favor downward perceived motion or “top-first” responses. Such visual motion biases are frequently reported yet are highly dependent on specific task parameters^53^. Thus, without further testing, it is difficult for us to conclude to what extent this played a role in NH response bias.

Interestingly, reduced response bias in the CI group also corresponded to group differences in vTOJ thresholds. Accuracy in this task has previously been shown to significantly decrease with age in typical individuals^33^. Not surprisingly, group differences were only evident when age was included as a covariate in the comparison to correct for the fact that the CI group was older by 8.4 years (Table 2). A preliminary study from our group also suggests that visual temporal thresholds in prelingually deafened adults with CIs are predictive of speech comprehension^54^. In the future, closer age-matching between groups, particularly with the vTOJ task, will better eliminate this potential confound and allow us to further investigate between-group differences.

Although aTOJ was equivalent for CI users and controls at threshold (Table 1), CI users seemed to exhibit lower performance across most SOAs (Fig. 6b) that was not evident in the global threshold measurements (Fig. 6c). Reduced accuracy as well as d′ across many SOAs (Fig. 7e) suggests that the frequency discrimination in this task may be comparatively more difficult for CI users. That is, lower performance even at the largest SOA does not suggest an auditory temporal processing deficit per se but rather a confounding factor of frequency discrimination inherent to the task (for a similar discussion, see REF: 53). Although 500 Hz and 2 kHz tones were detectable for CI users, we believe that the necessary discrimination overshadowed our ability to measure temporal processing by itself. In fact, this frequency component may also explain why we have consistently found auditory thresholds to be larger than visual thresholds with this task (Fig. 6c)^33,34,36^, despite the auditory system’s specialization for temporal processing. Although it is inconclusive on the basis of this task whether auditory temporal processing was intact in our CI cohort, several prior studies of gap detection thresholds indicate normal thresholds in CI users, particularly those with postlingual onset of deafness^55,56^. Furthermore, gap detection performance is known to approach adult-like thresholds by adolescence^57^, which in our cohort, would have occurred prior to severe-to-profound hearing loss. Thus, while we do not anticipate any auditory temporal impairments in the postlingually deafened CI users studied here, we cannot rule out this possibility.

A notable limitation to our interpretations of all signal detection analyses is that without having counterbalanced responses to different numbers, biases toward vision and audition, for instance, cannot be distinguished from preference for the numbers 1 or 2 (see instructions in Fig. 1). This could be a considerable confound when testing children; however, in adults who confirmed understanding of the task, such superficial biases seem less likely. Furthermore, future incorporation of reaction time measures into these tasks may further reveal perceptual differences with speeded judgments as others have shown in deafness^58^.

In conclusion, we show here that adult CI users judge the temporal relationship between auditory and visual speech in a visually-biased manner; however, the benefits (or consequences) of this weighting remain unknown. Ongoing work in our laboratory aims to elucidate how the present findings for temporal processing differences map onto the considerable clinical diversity in this population and has the potential to yield important insights—for example, into how the aforementioned results relate to AV integration of words, auditory-only speech recognition, and perhaps even broader language comprehension in CI users. Future investigations exploring the impact and generalization of temporal training^59–62^ will be beneficial for addressing whether remediation of altered temporal perception can positively impact AV gain for CI users. Ideally, this intervention could afford users with the maximum possible benefit from their CIs, which is closely tied with quality of life measures in this rapidly growing clinical population.

## Methods

### Participants

This study included 56 postlingually deafened CI users and 55 NH controls between the ages of 19 and 77 years old. Four participants (3 CI users, 1 NH control) were excluded from final analyses due to excessive missing data (i.e., for more than 50% of the tasks). Five additional participants (all CI users) were excluded due to: non-functional implants (n = 2), impaired vision (n = 1), and other confounding neurological diagnoses (n = 2). On average the NH controls (N = 54) were 8.4 years younger than CI users (N = 48; *t*_(1,100)_ = 2.8, *p* = 0.007; Table 2). As a result, age was included as a covariate for between-groups comparisons (see Results).

**Table 2:** Clinical and demographic characterization of cochlear implant (CI) and normal hearing (NH) groups.

Group	N	Sex (% female)	Mean age ± SD (y)	PTA (dB SPL)		Number of CIs	Acoustic hearing	Implant manufacturer	nonverbal IQ
				L	R				
CI	48	54%	53.4 ± 13.6*	26 ± 7	25 ± 6	1–60% 2–40%	48%	60% Cochlear 29% MED-EL 10% AB	102 ± 15*
NH	54	76%	45.0 ± 16.5	9 ± 7	10 ± 8	—	100%	—	109 ± 13

Pure Tone Averages (PTA) measure CI aided detection thresholds for the CI group; values are means ± standard deviation, *p < 0.01 CI = cochlear implant; NH = normal hearing; y = years; PTA = pure tone averages; dB SPL = decibels of sound pressure level; AB = Advanced Bionics.

In the control group, standard audiometric testing ensured normal pure tone averages (left ear 9 ± 7 dB, right ear 10 ± 8 dB) and speech perception (AzBio sentences, range 98–100% correct). We obtained aided audiograms at the study visit for the majority of CI users (n = 27) indicating that pure tone averages were appropriate for stimulus audibility (left 25 ± 6 dB, right 25 ± 6 dB). The remaining CI users (n = 21) were screened for detection at 30 dB at the time of testing. Speech perception in the CI group was measured via standard monosyllabic, consonant-nucleus-consonant (CNC) word lists, which indicated a wide range of proficiency consistent with other reports in the literature^1^.

CI users were required to have at least 3 months of experience with their implants prior to testing. The average experience was 3.5 years (range of 4 months to 11 years). The mini-mental state exam (MMSE) was also administered to screen for cognitive impairment defined by scores below 24 (of 30 possible points), and no exclusions were made based on this criterion (CI 29 ± 2; NH 29 ± 3). To minimize possible confounds with language, we focused on the nonverbal subscore of the Kaufman Brief Intelligence Test (KBIT) that indicated both groups had mean scores of nonverbal cognition within the age-normative range. There were, however, significant group differences where the controls scored slightly higher on average (CI = 102 ± 15; NH = 109 ± 13; *t*_(87)_= −2.1, *p* = 0.038).

All CI users were postlingually deafened and used the most current generation sound processors at the time of experimentation for all auditory testing. Additionally, all participants were screened for visual acuity by verbal confirmation and/or a Snellen eye chart, wearing corrective lenses as needed.

### Stimuli

Visual stimuli were generated in Matlab 2008a using Psychtoolbox extensions^63^. They were presented on a CRT monitor (100 Hz refresh rate) positioned approximately 50 cm from participants. Visual stimuli were white rings and circles (10 ms in duration) on a black background. Articulations of the syllables “ba” and “ga” were produced by an adult female speaking at a normal rate and volume with a neutral facial expression. Auditory stimuli were delivered at a comfortably loud level (calibrated to 65 dB SPL in the sound field) presented through stereo speakers. Auditory stimuli included tones (10 ms in duration) ranging from 500 Hz to 2 kHz as well as utterances of the syllables “ba” and “ga.” For flashbeep tasks, an oscilloscope (Hameg Instruments, HM407-2) was used to align auditory and visual stimuli to objective synchrony (0 ms) or the stimulus onset asynchronies (SOAs) shown in Fig. 1. For speech tasks, natural speech was considered objective synchrony.

### Procedures

All protocols and procedures were approved by Vanderbilt University Medical Center’s Institutional Review Board, and all methods were performed in accordance with these guidelines and regulations, which included all volunteers providing informed consent prior to participation. Experiments took place in a dimly lit, sound-attenuated room with an experimenter seated nearby. Both task order and trial order were pseudo randomized. On all non-speech tasks, subjects were instructed to maintain fixation on the centrally-located fixation cross. All responses were collected using a standard keyboard, and all testing was completed over 1 or 2 study visits.

### Analysis

For the unisensory TOJ tasks, we collapsed across positive and negative SOAs to plot accuracy at each temporal offset regardless of the stimulus position (for vTOJ) or frequency (for aTOJ). These data points were then fit with a standard logit function using the Matlab function glmfit to derive a threshold at 75% of the psychometric curve. For all tasks, any subject’s threshold that exceeded the largest SOA was excluded. Individuals who had accuracy higher than 75% at all tested SOAs were assigned a conservative threshold of the smallest SOA tested, which was 10 ms for both aTOJ and vTOJ tasks.

In all multisensory tasks, negative SOAs correspond to auditory-leading stimuli and positive SOAs correspond to visually-leading stimuli. Any bias in PSS is indicated by the sign, which typically reflects perceptual biases related to the slightly visually-leading onsets of natural speech (i.e., delayed voicing relative to orofacial articulations), adaptations to differing physical transmission times of light and sound, and differing neural processing times^24^. Because objective synchrony at 0 ms is the only point at which participants are truly “correct” in their simultaneity judgment, these tasks are unsuitable for further SDT analysis, so we focused this analysis on TOJ tasks where responses could be coded as hits (H) and false alarms (F).

We calculated d′ using the conventional formula of subtracting the z scores of the false alarm rate from the hit rate (d′ = z(H) − z(F)), with hits defined as correct “visual-first” responses and false alarms defined as incorrect “visual-first” responses. Put another way, for the AV TOJ tasks, as an example, we coded responses as if in response to the question “did the visual stimulus appear first?” such that “hits” were “yes” responses to positive SOAs (i.e., VA) and “false alarms” were “yes” responses to negative SOAs (i.e., AV). Next, we calculated a measure of response bias using the formula c = −0.5 × [z(H) + z(FA)]. It should be noted that in this circumstance, response bias is not a fixed characteristic of the observer, but instead shifts systematically with SOA. This results from the fact that we are not calculating criterion from a 0 ms SOA, which would indicate an individual’s fixed criterion. Instead, as the magnitude of the SOA increases, the proportion of correct “visual-first” responses, for instance, necessarily increases and the proportion of incorrect “auditory first” responses necessarily decreases. Confidence intervals at each SOA were calculated as discussed in MacMillan and Creel^64^.

For SJ tasks, mean reports of synchrony were plotted at each SOA, and the data were fit with two intersecting logit functions. All curves were normalized to 100% perceived simultaneity for TBW calculations, which was defined as the distance between the left and right curves at 75% reported synchrony. Simultaneity data were also fit with single Gaussian functions in R software (R code team, 2012) to derive the PSS at the peak of each curve.

SPSS Statistics for Macintosh Version 24.0 (IBM) and Prism 7.0b for Mac OSX (Graphpad Software) were used for statistical comparisons and graphing, respectively. All figures illustrate 95% confidence intervals of the mean. Otherwise, variance is indicated as standard deviation throughout the text.

### Statistical approaches

We utilized a non-parametric approach to evaluate between-group differences in audiovisual integration (i.e., bootstrapping). Missing data occurred for several reasons including insufficient testing time and more commonly, the inability to derive thresholds from curves for a variety of reasons such as participant fatigue, poor attention, misunderstood instructions, and insufficient SOA magnitudes. Missing data was handled by pairwise deletion.

Univariate regressions were carried out in initial tests of between-group differences in temporal processing across eight summary metrics (SJ speech PSS, SJ flashbeep PSS, SJ speech TBW, SJ speech PSS, aTOJ threshold, and vTOJ threshold, avTOJ flashbeep PSS, and avTOJ speech PSS). Given the aforementioned between-group differences in age and nonverbal IQ, these background variables were explored as covariates and retained in all models where they accounted for significant variance. Multivariate follow-up analyses were used to further characterize the nature of statistically significant between-group differences. When significant, further univariate tests of SOA-level differences were performed.
